# The dominant follicle: the final frontier in bovine oocyte development

**DOI:** 10.1590/1984-3143-AR2025-0071

**Published:** 2025-08-18

**Authors:** Lais Barbosa Latorraca, Antonio Galvão, Julietta Maria D’Augero, Gavin Kelsey, Noof Abdulrahman Alrabiah, Trudee Fair

**Affiliations:** 1 School of Agriculture and Food Science, University College Dublin, Dublin, Ireland; 2 Epigenetics Programme, The Babraham Institute, Cambridge, United Kingdom; 3 Department of Comparative Biomedical Sciences, Royal Veterinary College, London, United Kingdom; 4 Centre for Trophoblast Research, University of Cambridge, Cambridge, United Kingdom; 5 Wellcome-MRC Institute of Metabolic Science-Metabolic Research Laboratories, University of Cambridge, Cambridge, United Kingdom

**Keywords:** dominant follicle, oocyte competence, RNA sequencing, DNA methylation

## Abstract

The acquisition of oocyte competence in cattle, encompassing both cytoplasmic and nuclear maturation, is essential for successful fertilization and embryonic development. This competence is progressively achieved during the latter stages of the oocyte growth phase and completed within the dominant follicle (DF). The unique hormonal and immunological environment of the DF during oestrous supports oocyte “capacitation,” a process involving organelle reorganization, mRNA storage and meiosis resumption, which fully prepares the oocyte for fertilization. These changes differentiate oocytes from the DF from those of subordinate follicles, explaining why only oocytes from the DF mature and ovulate successfully. Despite advances in assisted reproductive technologies like in vitro maturation (IVM) and in vitro fertilization (IVF), developmental outcomes remain inferior compared to in vivo matured oocytes, largely due to incomplete or altered oocyte maturation *in vitro*. Blastocyst rates after IVM/IVF are substantially lower (~35%) than those from *in vivo* matured oocytes (58-78%). The heterogeneity of oocytes retrieved from antral follicles and the lack of exposure to the natural follicular environment during IVM are key factors limiting developmental competence. Here we describe the molecular changes in bovine oocytes from DFs, collected at 24 and 2 h before ovulation without ovarian stimulation, using single-cell RNA sequencing and bisulfite sequencing to assess gene expression and DNA methylation dynamics. Results revealed significant shifts in transcripts related to oxidative phosphorylation, highlighting the crucial role of energy metabolism during oocyte capacitation. DNA methylation changes were subtle but indicated a more dynamic and less stable epigenome in fully-grown oocytes than previously assumed. Overall, understanding the gene expression and epigenetic landscape during oocyte maturation in the DF offers valuable insights into improving oocyte quality and ART outcomes in cattle. Optimizing the maturation environment to better mimic natural follicular conditions could enhance reproductive efficiency in bovine production systems.

## Introduction

The development of mammalian oocytes and follicles is characterised by a progressive series of molecular and morphological changes ultimately leading to a competent oocyte capable of being fertilized and supporting embryonic development for a review see (Fair, 2010; Fair and Lonergan, 2023; Hyttel et al., 1997). The final period of oocyte development within an antral follicle is particularly critical for the acquisition of oocyte competence and involves cessation of transcription, organelle reorganisation, mRNA storage, and in an ovulatory follicle, the resumption of meiosis (Fair and Lonergan, 2023; Telfer et al., 2023). Oocyte competence is dependent on completion of cytoplasmic and nuclear maturation. Cytoplasmic maturation involves the reorganisation of the cytoskeleton and organelles (Assey et al., 1994; Hyttel et al., 1997), a process orchestrated by microtubules and microfilaments (Li and Albertini, 2013). Nuclear maturation, begins with the resumption of meiosis I, characterised by germinal vesicle breakdown (GVBD) and proper chromosome alignment during spindle formation, critical for normal chromatin segregation and progression to metaphase II (Harasimov et al., 2023). The maturation environment has the greatest impact on developmental outcome, for example, in vitro maturation (IVM), is the rate limiting step in embryo production in vitro (IVP). Irrespective of the embryo culture environment, blastocyst development rates of *in vivo* matured oocytes are far higher than those of oocytes derived by IVM and IVF (Lonergan and Fair, 2008). The heterogeneity of the population of aspirated antral follicle oocytes appears to be the key contributor to the disparity in success of oocytes aspirated from antral follicles and submitted to IVP, compared to those matured and fertilized *in vivo* (Sirard and Blondin, 1996; van Blerkom, 1990). Fully competent oocytes are believed to have achieved ’cytoplasmic maturation’ (Barnes, Sirard, 2000), or been exposed to the capacitating actions of the dominant follicle (DF) (Hyttel et al., 1997).

In general, cattle have a 21-day oestrous cycle characterised by 2-3 waves of follicle growth. During each wave one follicle outgrows the others. The term follicle dominance was coined to describe the first day of the oestrous cycle that the largest follicle in a wave is at least 1 to 2 mm larger than the next largest follicle and subordinate follicles in the same wave cease growing. (Mihm et al., 1997; Sunderland et al., 1994). Follicle diameter deviation occurs when the follicles reach ~8.5 mm (Adams et al., 2008; Gomez-Leon et al., 2023). During the bovine oestrous cycle, only oocytes from the DF undergo maturation, resulting in the ovulation of one competent oocyte per cycle. The ultrastructure of the nucleus and cytoplasm of oocytes retrieved from developing DF is significantly modified compared to that of oocytes from subordinate follicles (Assey et al., 1994). The term “oocyte capacitation” was suggested to reflect that the oocyte reaches its full capacity during this phase of development (Hyttel et al., 1997), a phase that most oocytes submitted to IVM/IVP do not experience.

Understanding the dynamic changes occurring in the DF and the COC within, is crucial for improving outcomes for assisted reproduction technologies (ART). Here we highlight key features and discuss their potential contribution to oocyte developmental competence.

## Heterogeneity of antral follicle oocytes

Oocyte quality determines successful fertilisation and development to the blastocyst stage. Despite their value in genetic breeding programmes and continuous refinement, blastocyst and pregnancy rates in IVP systems remain low, with oocyte maturation identified as a critical bottleneck (Jiang et al., 2023; Lonergan and Fair, 2016; Luciano and Sirard, 2018). Many research laboratories base their studies on oocytes aspirated from 3 to 6 mm follicles on ovaries collected at local abattoirs, which coupled with oocyte pooling, makes tracking the origin of analysed oocytes difficult. Moreover, the blastocyst development rates of 2-cell embryos derived by IVM/ IVF (Hoelker et al., 2017; Rizos et al., 2002) plateau at 35-40% compared to 58 to 78% from *in vivo* matured oocytes. Factors such as donor age, follicle diameter, stage of oocyte growth/oocyte diameter and COC morphology all impact the oocyte’s ability to achieve competence (see thorough review (Walker and Biase, 2020). Even when oocytes are selected for IVP from similar-sized follicles, variation in their developmental competence has long been recognised (Sirard and Blondin, 1996; van Blerkom, 1990), due to the prevalence of atresia among most of these follicles (Kruip and Dieleman, 1982). Many molecular analyses of oocytes, cumulus cells, follicular fluid, theca and granulosa cells describing factors correlated with competence have been published. Much of this research involves pools of oocytes aspirated from 3 to 6 mm follicles on ovaries sourced at local abattoirs, therefore tracking the origin of analysed oocytes and elucidating physiologically relevant information is difficult (Walker and Biase, 2020). In general, most studies show that oocytes collected from larger follicles produce more blastocyts *in vitro* (Blondin et al., 1997; Lonergan et al., 1994). Nevertheless, access to rapidly evolving single cell transcriptomic, proteomic and metabolomic technologies is enabling great strides in our understanding; a modified single-cell multi-omics approach was employed recently to analyse the transcriptome, DNA methylome and chromatin accessibility of individual oocytes and their cumulus cells collected from small (<3 mm), medium (3-8 mm), and large (>8 mm) bovine antral follicles and compared to *in vivo* derived MII stage oocytes (Zhang et al., 2024). Interestingly, correlation analysis of the transcriptomic data showed that oocytes from large follicles were more similar to *in vivo* metaphase II oocytes than those from small and medium follicles. Moreover inflammation, DNA damage and p53 signaling pathways were enriched in oocytes from the smallest follicles. The Authors reported little difference in oocyte diameter regardless of follicle size, suggesting that all oocytes had more or less completed their growth phase. Considering the greater developmental potential of oocytes from larger follicles and an earlier report of upregulated expression of inflammatory pathways in atretic follicles compared to healthy follicles (Hatzirodos et al., 2014), one could speculate that most small follicles and the oocytes within are atretic, highlighting again the value of interrogating the external and internal environment of the DF and it’s impact on the molecular profile of the COC within, to refine ART protocols and media.

## Endocrine environment of the dominant follicle

Hormonal regulation of the oestrous cycle is dependent on a positive and negative feedback circuit between the hypothalamus, the anterior pituitary, the ovaries and the uterus. Gonadotrophin-releasing hormone (GnRH) produced and released by the hypothalamus regulates the secretion of the gonadotrophs, follicle-stimulating hormone (FSH) and luteinising hormone (LH) from the anterior pituitary (Schally et al., 1971). Two to three waves of follicle growth are stimulated by a transient rise in FSH per oestrous cycle, while the subsequent decline in FSH stimulates development of the DF during a wave (for review, see (Ireland et al., 2000), such that each wave is characterised by follicle emergence, selection and dominance. The regression of the corpus luteum (CL) in response to prostaglandin (PGF) secretion from the uterus (Hansel and Echternkamp, 1972), during the follicular phase of the oestrous cycle leads to basal systemic progesterone (P4). The parallel rapid growth of and increasing estradiol (E2) secretion by -the pre-ovulatory DF induces a surge in GnRH which in turn induces a coincidental LH and FSH surge (Sunderland et al., 1994). The DF ovulates when serum P4 levels are basal and LH pulses occur every 40-70 min for 2-3 days (Channing et al., 1980). However, within the DF, follicular fluid P4 concentration almost trebles following the LH surge increasing from~ 60ng/mL to ~170ng/ml (Abdulrahman Alrabiah et al., 2021). Following ovulation, the CL forms from the ovulated follicle, the granulosa and theca cells of the ovulated DF luteinise and produce P4 (Smith et al., 1994). While recurrent waves of follicle development continue during the luteal phase of the oestrous cycle, the DF does not ovulate due to the suppressive negative feedback effect of P4 on the frequency of LH pulses (Gomez-León et al., 2020). Progesterone during the growth of the ovulatory follicle is important for oocyte quality, the first follicular wave is characterised by low circulating P4 output by the developing CL, therefore first wave ovulations are associated with the recovery of less transferable embyros following superstimulation (Nasser et al., 2011; Rivera et al., 2011) and lower pregnancies per AI in lactating dairy cows (Denicol et al., 2012), which were overcome by intravaginal P4 supplementation.

Oestradiol concentrations are higher in the follicular fluid of the DF compared to that of subordinate follicles of the same wave (Fortune et al., 2001; Ireland and Roche, 1983; Mihm et al., 1997; Sunderland et al., 1994). The concentration increases as DF growth continues, reaching concentrations greater than 1 μg/mL in preovulatory follicular fluid (Fortune and Hansel, 1985). The synthesis of E2 is dependent on the production of androgens by theca cells which diffuse into granulosa cells where they are aromatised (Fortune and Quirk, 1988). During antral follicle development the granulosa cells of the follicle undergoing selection express LH receptors (Ireland and Roche, 1982). Following the LH surge, granulosa cell LH receptor binding of LH is pivotal to the induction of ovulation, initiating a switch from E2 to P4 production within 6 h of oestrus (Dieleman et al., 1983), propagating the EGF-signaling cascade essential to oocyte resumption of meiosis and the formation of the CL (for review see (Richani and Gilchrist, 2018). During the final 24 h before ovulation, the ratio of E2:P4 dramatically changes from 45:1 to almost 1:1 (Fortune and Hansel, 1985). This profound change in follicular fluid steroid concentration during the critical period of oocyte maturation may influence the subsequent competence of the ovulated oocyte to support fertilization and early embryo development (Aardema et al., 2013; Fair and Lonergan, 2012)

Both the health status and the metabolic status of the cow may alter the steroidogenic capacity of the DF by affecting cholesterol transport into the mitochondria to initiate steroidogenesis, evidenced by reduced systemic E2 and P4 concentrations in cows with uterine disease (Seekford et al., 2025) and in lactating cows compared to non-lactating dairy heifers and in nutrient restricted beef heifers compared to their counterparts on a non-restricted diet (Walsh et al., 2012a). The metabolic response of cows to milk production varies for many reasons, some animals experience severe loss of body condition in the early post-partum period. A recent study reported lower IGF1 and E2 concentrations in the follicular fluid at 7 weeks after calving along with reduced granulosa cell mRNA expression of genes associated with DF competence, including *CYP19A1*, *NR5A2*, *IGF1*, and *LHCGR*, in the DF of a synchronised follicular wave in cows that experienced a 1 point loss in body condition score compared to cows that sustained a more moderate loss in condition (Alemu et al., 2024). Similarly, exposure of high yielding dairy cows to summer heat stress during the early post-partum period can aggravate body condition loss resulting in smaller DF with lower follicular fluid glucose, IGF-1, NEFA, urea and total cholesterol concentrations (Shehab-El-Deen et al., 2010). The bioavailability of IGF-1, was previously associated with the growth, proliferation and steroidogenic capacity of the future DF (Canty et al., 2006; Mihm et al., 2000). Strong negative correlations between follicular fluid concentrations of E2 and the low MW IGF-binding proteins (IGFBP) suggest their potential role in regulating intrafollicular availability of free IGF1, (Spicer, 2004).

## Immune cells and cytokine profile

The importance of the immune system as a key mediator of ovarian function was recognised several decades ago (Espey, 1980). Within hours of the pre-ovulatory LH surge, the modest population of resident leukocytes in the ovary expands dramatically as leukocytes, primarily from circulating blood and the spleen, invade the ovary. In rats, the scale of the invasion was quantified as a doubling of the ovarian leukocyte population (Oakley et al., 2010). Infiltrating leukocytes are involved in many different aspects of ovulation; from amplifying inflammatory signals, follicular wall degradation, stimulating tissue remodelling and facilitating tissue repair post ovulation (Duffy et al., 2019; Fair, 2015). Pre and peri-ovulatory bovine follicles host a broad repertoire of immune cells, including T-cells, granulocytes and monocytes, moreover CD11c-CD172a+ cells, previously described as monocyte-derived dendritic cells (MoDC) (Palmer et al., 2009), were localized in the theca layers of bovine pre- and peri-ovulatory follicles in extraordinarily high numbers (>350 cells/mm^2^) (Abdulrahman Alrabiah et al., 2021). Previously, ablation of CD11c+ cells by diphtheria toxin administration to CD11c-diphtheria toxin receptor transgenic mice indicated a pivotal role for putative MoDC in the ovulation process (Cohen-Fredarow et al., 2014). More importantly, the Authors reported that cumulus oocyte complexes were unexpanded, showed limited mucification and remained trapped within luteinizing follicles and inflammation-associated genes were significantly upregulated in ovarian tissue.

In parallel with immune cell profiling of the bovine pre- and peri-ovulatory follicles, we simultaneously quantified the concentration of multiple cytokines in the follicular fluid (Abdulrahman Alrabiah et al., 2021). While the concentrations of several cytokines fell below the sensitivity of the assay, CXCL10, VEGFA, IL10, IL36RA, CCL2, CCL3, CCL4 and IFNG were detected and quantified. Of note, IL10 and VEGF-A represented the most concentrated cytokines in pre-ovulatory and CXCL10 in peri-ovulatory -follicular fluids. It is likely that the large population of MoDC recruited to the ovulatory follicle is the source of CXCL10. The concentrations of CCL2, CCL3 and CCL4 were notably high; they interact with the neutrophil expressed receptors, CCR1, (CCL3 and CCL4, (Sanz and Kubes, 2012)) and CCR2 (CCL2, (Johnston et al., 1999)), and are therefore likely to guide neutrophil recruitment to the DF prior to the LH surge (Abdulrahman Alrabiah et al., 2021). *VEGF* is expressed by theca, granulosa and luteal tissue cells (Abdulrahman Alrabiah et al., 2021; Chowdhury et al., 2010; Stouffer et al., 2007; Walsh et al., 2012b). It is chemotactic for monocytes, macrophages and endothelial cells and plays a regulatory role in angiogenesis (Ferrara and Davis-Smyth, 1997). Monocytes, macrophages and neutrophils produce VEGF (Gargett and Rogers, 2001; McLaren et al., 1996), and macrophage VEGF production is upregulated in response to hCG or LH (Guimerà et al., 2009). Thus it is likely that the endocrine and immunological conditions within the DF amplify VEGF synthesis in the DF/ ovulatory follicle where it’s key role is likely in CL formation (see reviews by (Reisinger et al., 2007; Shirasuna et al., 2013). It is also interesting to note that human follicular fluid VEGF-A and CXCL-6 concentration was reported to strongly correlate with oocyte maturity from the mid-antral to preovulatory stage and could be used in combination to predict oocyte maturity during IVF (Chen et al., 2023).

## Metabolomic constituents

An appropriate biochemical environment is essential to optimal oocyte development and maturation in vivo or in vitro. Follicular fluid metabolites include the hormones, growth factors and cytokines highlighted above and include amino acids, lipids, carbohydrates, nucleotides, and other small molecules derived from serum and the metabolic activity of granulosa, theca and immune cells in the DF/peri-ovulatory follicle (Fortune et al., 2004; Gosden et al., 1988). They are also proposed to protect the oocyte against proteolysis and provide the necessary intracellular metabolites for ovulation (Da Broi et al., 2018). The modulation of follicular fluid constituents during follicle development suggests a progressive adaptation to provide the appropriate microenvironment to promote oocyte quality and subsequent developmental competence (Bender et al., 2010; Forde et al., 2016; Leroy et al., 2011; Matoba et al., 2014). This is particularly important during the peri-ovulatory period when the energy requirements to drive oocyte meiotic resumption and maturation must be met (Dumesic et al., 2015). Engaging high throughput untargeted liquid chromatography tandem mass spectroscopy, we identified over 600 metabolites in pre- (24 h) and peri- (2h) ovulatory follicular fluid, comprising: lipids (37.1%), amino acids (30.0%), xenobiotics (11.5%), nucleotides (6.8%), carbohydrates (4.4%), cofactors and vitamins (4.4%), peptides (3.6%) and energy substrates (2.1%) (Abdulrahman Alrabiah et al., 2023). The qualitative metabolomic profiles were 99% identical, however 10% of total metabolites were significantly modulated during the final 24 h ovulatory period. The differences were primarily due to flux in lipid (43.3%) and amino acid (28.4%) concentrations with some dramatic change in a few metabolites: Hypoxanthine and xanthine exhibited the greatest reduction (98.9- and 65.7- fold, respectively), while retinal, 1-methyl-5-imidazoleacetate and isovalerylcarnitine (4.9, 2.7 and 2.7 -fold, respectively), the greatest corresponding increases. Hypoxanthine is an established inhibitor of the resumption of oocyte meiotic maturation in cattle (Kadam and Koide, 1990) and mice (Downs et al., 1986; Eppig et al., 1985), and has also been identified in human, porcine and caprine follicular fluid (Lavy et al., 1990; Ma et al., 2003; Miyano et al., 1995, respectively). The depletion of hypoxanthine and associated compounds in follicular fluid by 2 h pre-ovulation reflects the culmination of LH activated signaling cascades within the preovulatory follicle leading to the release of the oocyte from meiotic arrest.

Polyunsaturated fatty acids (PUFA) are bioactive lipids with immunomodulatory properties; ω-3 PUFA and ω-6 PUFA are generally considered anti-inflammatory and pro-inflammatory, respectively (Michalak et al., 2016), and were jointly the most abundant metabolite. In cattle, follicular fluid ω-3 PUFA α-linoleic acid levels are associated with oocyte competence to form blastocysts in vitro (Matoba et al., 2014), possibly due to scavenging reactive oxygen species (ROS) ((Marei et al., 2012). Other stable constituent antioxidants identified in FF include carotenes, glutathione, urate, and ascorbic acids (Vitamin C). Polyunsaturated fatty acids are precursors to prostaglandin (PG) synthesis, particularly arachidonic acid which may be converted by granulosa cells to PGE2 and PGF2α (Algire et al., 1992). The synthesis of proinflammatory PG begins 18-24 h after GnRH administration (Bridges et al., 2006), prostaglandins E2 (PGE2) and F2 alpha (PGF2α) were first detected in FF at 2h pre-ovulation (Abdulrahman Alrabiah et al., 2023). The fine balance of controlling inflammation yet maintaining an immunoactive environment within the pre-ovulatory follicle is further exemplified by a parallel increase in retinal (4.9-fold). Retinal is one of three Vitamin A (or retinoid) forms, retinoids are generally considered anti-inflammatory (Huang et al., 2011), they act on cells of both the innate and adaptive immune systems (Oliveira et al., 2018). It is presumed that granulosa cells take up retinol and convert it to retinal and retinoic acid (RA) within the follicle (Liu et al., 2018), where it may act as an antioxidant (Ikeda et al., 2005), and or contribute to the regulation of steroidogenesis (for review see (Damdimopoulou et al., 2019). Supplementation of oocyte IVM medium with RA improved bovine IVP blastocyst development rates (Lima et al., 2006; Livingston et al., 2004), possibly associated with improved oocyte meiotic maturation (Gad et al., 2018). Metabolomic data sets provide a rich resource for refinement of oocyte or follicle *in vitro* culture systems.

## Transcriptome and DNA methylome landscape

Advanced RNA and DNA sequencing technologies have become indispensable to multi-omic approaches to explore follicle and oocyte genomics under various conditions. RNA seq analysis of the temporal changes in dominant follicle theca and granulosa cell gene expression at distinct stages of dominant follicle development, including selection, differentiation and the peri-ovulatory period revealed dramatic changes in the transcriptomes of both tissue types at each stage (Walsh et al., 2012a). In particular, genes within the biosynthesis of steroids pathway and genes reflective of the dynamic flux in the immune cell population during DF differentiation and luteinisation were temporally expressed in the transcriptomes of theca and granulosa cells (Walsh et al., 2012a, b); immune pathways related to leucocyte extravasation and chemotaxis were over-represented in theca cells, whereas immune pathways related to inflammation and innate immune response were over-represented in granulosa cells. Notably, both profiles were influenced by the physiological status of lactation, highlighting the potential contribution of an impaired inflammatory process during ovulation to compromised peri-ovulatory follicle function in metabolically challenged animals (Walsh et al., 2012b). Much more recently, Tariq et al. (2025), demonstrated the negative impact of IVM in the presence of lipopolysaccharide (LPS) or granulosa cell and LPS conditioned media, on bovine oocyte developmental competence and subsequent embryo quality.

We recently carried out single-cell RNA-sequencing of GV-stage bovine oocytes of known diameters (<60 to >120 μm), bioinformatic analysis of the data identified three particularly noteworthy clusters of co-expressed genes correlated with oocyte size. The first cluster of genes were positively correlated with oocyte size, i.e., their expression increased during oocyte growth, the genes in this cluster enriched response to stimulus, cell communication and negative regulation of signaling pathways. The second, comprised genes whose expression was negatively correlated with oocyte size and was populated by genes related to cell development, regulation of cell-matrix adhesion and electron transport chain. Interestingly, a cluster of 2,000 genes maintained constant expression during oocyte growth; they preferentially enriched pathways associated with establishment of organelle localisation, protein catabolic process, response to decreased oxygen levels, nuclear envelope organisation and glycosylation. Further analyses of the dataset identified a clear profile of decreased expression of genes associated with oxidative phosphorylation and increased expression of maternal genes and transcription regulators across the bovine oocyte growth phase. An interesting switch in gene expression profile was noted in oocytes greater than 100 μm in diameter, when the expression of genes related to cytoplasmic activities was replaced by genes related to nuclear activities (e.g., chromosome segregation). The most profound change in the molecular profile of oocytes was seen at the end of the oocyte growth phase, highlighting the importance of this final phase of oocyte growth to oocyte acquisition of competence (Latorraca et al., 2024).

## Transcriptome and methylome in oocytes from dominant follicles

Transcription activity decreases as the oocyte reaches the fully-grown stage (Fair et al., 1996), reflected in the restructuring of the nucleolus into an inactive dense fibrillar sphere surrounded by condensed chromatin and the migration of the cytoplasmic organelles towards the oocyte cortex (Fair et al., 1997; Labrecque et al., 2015). However, final acquisition of developmental competence or ‘oocyte capacitation’ occurs in the DF (Hyttel et al., 1997), simultaneous or, because of the dramatic changes in the DF’s endocrine, immunological and metabolic environment. This pre-maturational sequence of morphological changes includes expansion of the lipid compartment and reduction in the size of Golgi complexes in the cytoplasmic compartment and undulation of the nuclear envelope. Most interestingly, the nucleolus becomes vacuolated, developing into a ring-like structure where the fibrillar centre forms a distinct portion of the ring (Assey et al., 1994). The significance of nucleolus vacuolisation in the dominant follicle oocyte has been considered, on the one hand it may represent the dispersal of the nucleolus associated with resumption of prophase 1, while on the other, it could reflect a temporary resumption in transcriptional activity in the oocyte nucleus (Hyttel et al., 1997). This hypothesis is based on the similarity to the structure of the nucleolus in the 4-cell embryo, where vacuolisation of the nucleolus is associated with the first presumptive rRNA synthesis (Kopecný et al., 1989; Viuff et al., 1996; Viuff et al., 1998). While earlier studies have demonstrated that COCs require a 1-2 h period of transcription at the initiation of IVM in order to synthesise the proteins necessary to drive meiosis (Hunter and Moor, 1987; Kastrop et al., 1991), to date the most conclusive evidence indicates this transcription occurs in the corona cells and is trafficked into the oocyte via gap junctions between the cumulus process endings and the transzonal projections of the oolemma (Macaulay et al., 2016).

To determine if the DF modulates the transcriptomic and methylomic profile of the oocyte within, single-cell RNA and DNA methylation analysis was carried out on oocytes collected from synchronised non FSH-stimulated DF and periovulatory follicles collected during previously published animal trials performed at University College Dublin (Abdulrahman Alrabiah et al., 2021; Sánchez et al., 2024). The resulting data was subsequently compared to that of oocytes at the end of the growth phase (>120 μm in diameter), retrieved from the ovarian cortex by slicing, which was recently published within a transcriptomic profile of the bovine oocyte growth phase (Latorraca et al., 2024). The details of oocyte collection, single-cell RNA sequencing & bisulphite conversion and DNA methylation analysis are described in Supplementary File 1 (Supplementary Material).

**Figure 1:**
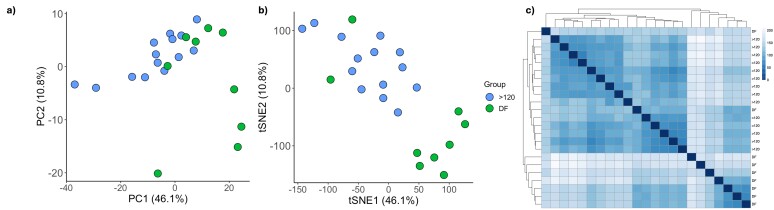
Sample distribution and variation. (a) PCA and (b) tSNE plots showing the distribution and clustering of >120 μm (blue) and dominant follicle (DF) (green) oocytes; (c) The heatmap shows sample variation within groups. Oocytes >120 μm in diameter oocytes were collected by slicing the ovarian cortex of abattoir derived ovaries; DF oocytes were collected from post-mortem oestrous synchronised animals around the time of ovulation.

**Figure 2:**
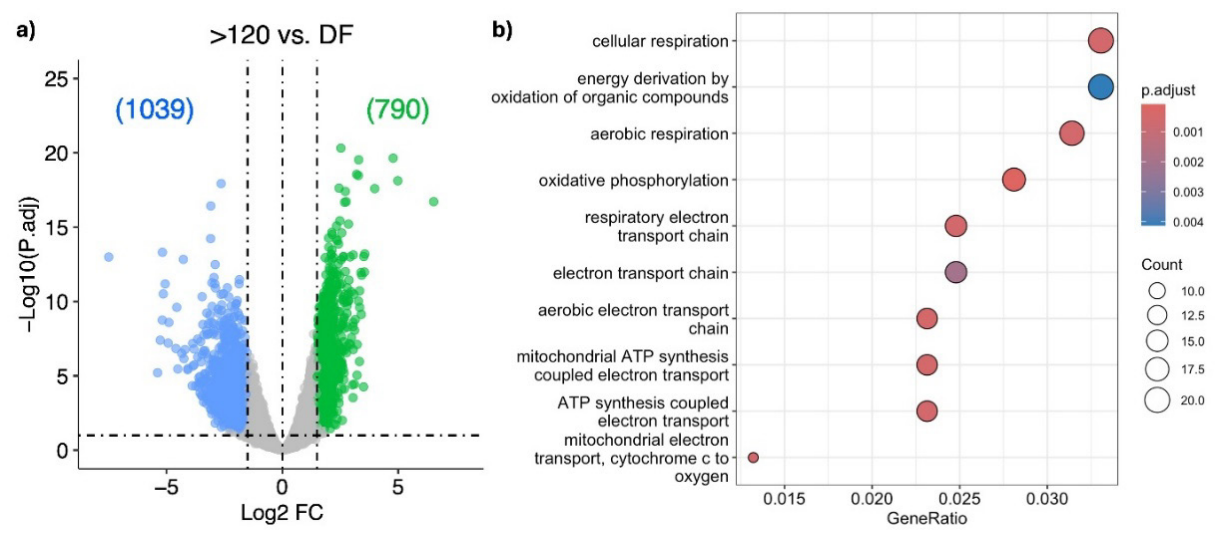
Differential expression analysis. (a) Vulcano plot showing the upregulated genes in each differentially expressed gene (DEG) group comparison (FDR-padj<0.05, log2FC>|1.5|); (b) Enriched pathways (FDR<0.05) populated by upregulated genes in DF oocytes compared to >120 μm oocytes.

The number of expressed genes (total counts higher than 1 for each group) was similar between groups (13,912 for >120 μm oocytes and 11,273 for DF oocytes), see Supplementary Table S1. Using dimension reduction and the sample distance matrix we observed higher variance in expression profiles of DF oocytes and greater clustering of expression profiles in oocytes >120 μm (Figure 1). Further filtering to exclude very low-abundant genes from the combined total of 15,975 expressed genes, resulted in a final list of 8,414 genes. In line with the decline in transcription in fully-grown oocytes, differential expression analysis between groups identified 1,039 genes in oocytes >120 μm oocytes and 790 genes in DF oocytes, whose expression was significantly higher, see Supplementary Table S2. Gene ontology analysis correlated upregulated genes with carbohydrate catabolic process with >120 μm oocytes and oxidative phosphorylation with DF oocytes (Figure 2). Thus, oxidative phosphorylation appears to be suppressed during oocyte growth and reactivated during the DF and peri-ovulatory period for ATP production to meet the future energy requirements of oocyte meiotic maturation, fertilisation and the subsequent early embryonic cleavage divisions. Although the number of expressed genes was similar between the two contrasted oocyte groups, they differ from findings related to oocytes matured *in vitro* (Mamo et al., 2011; Reyes et al., 2015). Transcript abundance of genes associated with cell cycle and cytoskeleton organisation was greater and those related to mitochondria organization and ribonucleoprotein complex biogenesis were reduced in MII oocytes compared to GV (Reyes et al., 2015). Since transcription has ceased prior to GVBD, differences in transcript abundance are likely explained by dynamic changes in mRNA processing, such as increased transcript poly-adenine tail length (Mamo et al., 2011), mRNA storage and degradation during maturation and processing during extraction (Fair and Lonergan, 2023). While *in vitro* systems might induce artificial shifts in the oocyte transcriptome, the importance of the quality of the starting material was elegantly demonstrated in a recent single-cell methylome and transcriptome sequencing analysis of oocytes from the same cow, either matured *in vivo* or *in vitro*, which reported very modest changes in gene expression (Benedetti et al., 2025). Regardless of final maturation environment, all the oocytes in that study had completed their growth phase within an optimised hormonal environment using an oestrus synchronisation protocol. Raw data are deposited in the Gene Expression Omnibus repository and are accessible through GEO accession number GSE297927.

**Figure 3:**
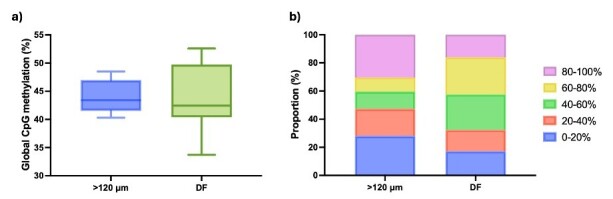
DNA methylation dynamics in fully-grown bovine oocytes. (a) Box plot showing global CpG methylation in oocytes >120 μm in diameter and oocytes collected from dominant follicles (DF); (b) Proportion of 50-CpG windows with 0-20%, 20-40%, 40-60%, 60-80%, and 80-100% methylation in oocytes >120 μm and DF oocytes.

**Figure 4:**
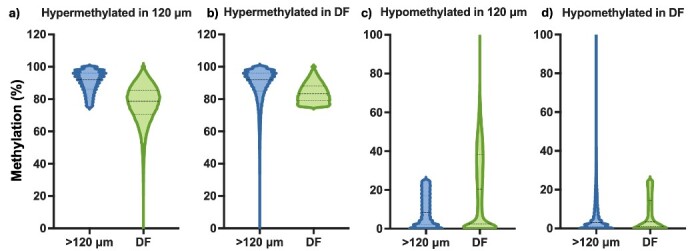
Methylation dynamics of hypermethylated 50-CpG windows quantified in >120 μm and DF oocytes. (a) Hypermethylated (>75%) windows in >120 μm oocytes; (b) Hypermethylated windows in DF oocytes; (c) Hypomethylated (<25%) regions in >120 μm oocytes; (d) Hypomethylated windows in DF oocytes.

**Figure 5:**
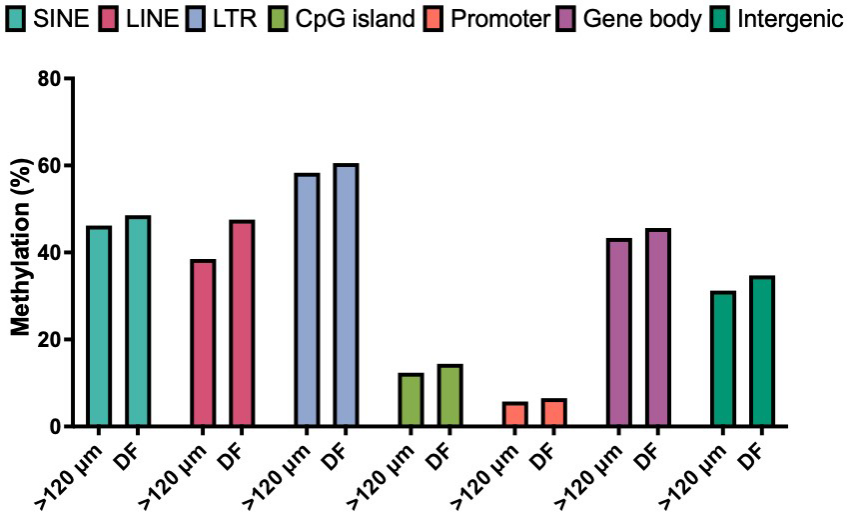
Methylation dynamics of bovine oocytes from dominant follicles. Bar graph showing the methylation levels of genomic features including SINEs, LINEs, LTRs, CpG islands, promoters, gene bodies, and intergenic regions. SINE: short interspersed nuclear elements; LINE: long interspersed nuclear elements; LTR: long-terminal repeats.

Epigenetic modulation also plays an important role in oocyte acquisition of competence, bisulphite sequencing was performed in parallel with mRNA seq analysis (the sequencing output for DNA libraries is summarized in Supplementary Table S3); global CpG methylation ranged from 44.11% in >120 μm oocytes to 43.81% in DF oocytes (Figure 3). In contrast to the similar average global CpG methylation, when the data were quantified over 50-CpG windows the pattern of hypomethylated, intermediate, and hypermethylated regions was different in the two oocyte groups: DF oocytes were characterised by decreased proportions of hypo and hyper-methylated windows and increased intermediate methylation (Figure 3). Interestingly, when selecting hypermethylated and hypomethylated 50-CpG windows in one group, we observed an almost opposite profile in the other group (Figure 4). For example, hypermethylated windows in >120 μm oocytes tended to lose methylation in DF oocytes. Similarly, hypermethylated DF windows also presented a higher methylati on variation in >120 μm oocytes; however, in this case, the average was higher in the second group. Furthermore, hypomethylated regions in >120 μm oocytes had a slightly higher average methylation in DF oocytes, with a few windows presenting hypermethylation levels. A similar pattern was observed by selecting DF hypomethylated windows; however, with a comparable average methylation level (Figure 4). A target analysis over genomic features showed a slight increase in the average methylation of LINEs, SINEs, LTRs, CpG islands, gene bodies, and intergenic regions in DF oocytes compared to >120 μm oocytes. Furthermore, transposable elements, gene bodies and intergenic regions presented higher levels of methylation compared to CpG islands and promoters, in agreement with previous findings (Ivanova et al., 2020) (Figure 5). The influence of the DF environment on the oocyte is further highlighted by Benedetti et al (Benedetti et al., 2025) in their single-cell analysis of oocytes from the same cow, who described hypermethylation in the whole-genome profile, CpG islands and gene bodies of in vivo matured oocytes compared to oocytes aspirated from 3-8 mm follicles and matured in vitro. Taken together, these findings agree with a recent report of ongoing methylation modifications in fully grown oocytes, albeit originating from small, medium and large bovine follicles (Zhang et al., 2024); a topic of debate in the literature (Demond and Kelsey, 2020; Sendžikaitė and Kelsey, 2019). Moreover, there appears to be some discrepancy in the published data, as one study reported higher global methylation (~40%) in DF oocytes from synchronised and stimulated donors compared to GV and in vitro MII oocytes from slaughterhouse ovaries (~30%), the authors also reported a prevalence of tiles with 0-20% methylation in GV, in vitro and in vivo MII oocytes (Jiang et al., 2018), while another reported a global methylation of 30% and the prevalence of regions with 40-60% methylation in similar samples (Duan et al., 2019). The differences are likely due to sample origin and sequencing techniques.

## Conclusion

Bovine DF development is orchestrated by a finely tuned hormonal feedback system which plays a pivotal role in the final stages of oocyte maturation and ovulation. The innate immune system is intricately involved in ovulation, marked by a neutrophil influx around the LH surge, followed by expansion of macrophage and dendritic cell populations within the follicle. This immune cell activity is tightly regulated, with simultaneous expression and synthesis of pro- and anti-inflammatory cytokines producing a controlled sterile inflammatory response that resolves within 24 hours as the CL forms. Differentially regulated metabolites, particularly those linked to inflammatory and immune responses, also modulate follicular inflammation and help maintain cellular homeostasis, balancing oxidants and antioxidants, thereby shaping the microenvironment enclosing the oocyte.

Consistent with the cessation of transcription at the end of the oocyte growth phase, bovine oocytes from DF exhibit limited changes to their transcriptome compared to fully-grown oocytes from small antral follicles in the ovarian cortex. However, in contrast to their down regulation during the oocyte growth phase, genes involved in oxidative phosphorylation are upregulated during this final period of development, highlighting the importance of energy metabolism during oocyte capacitation, likely to meet the increased energy demands associated with resumption of meiotic maturation, fertilisation and early embryonic development. The epigenome and transcriptome of fully-grown bovine oocytes from DFs are not entirely stable as even with the difficult access to chromatin due to its condensed configuration in fully-grown oocytes, cytosine methylation may be modified, as demonstrated by the methylation differences between the collected oocytes.

**Figure 6:**
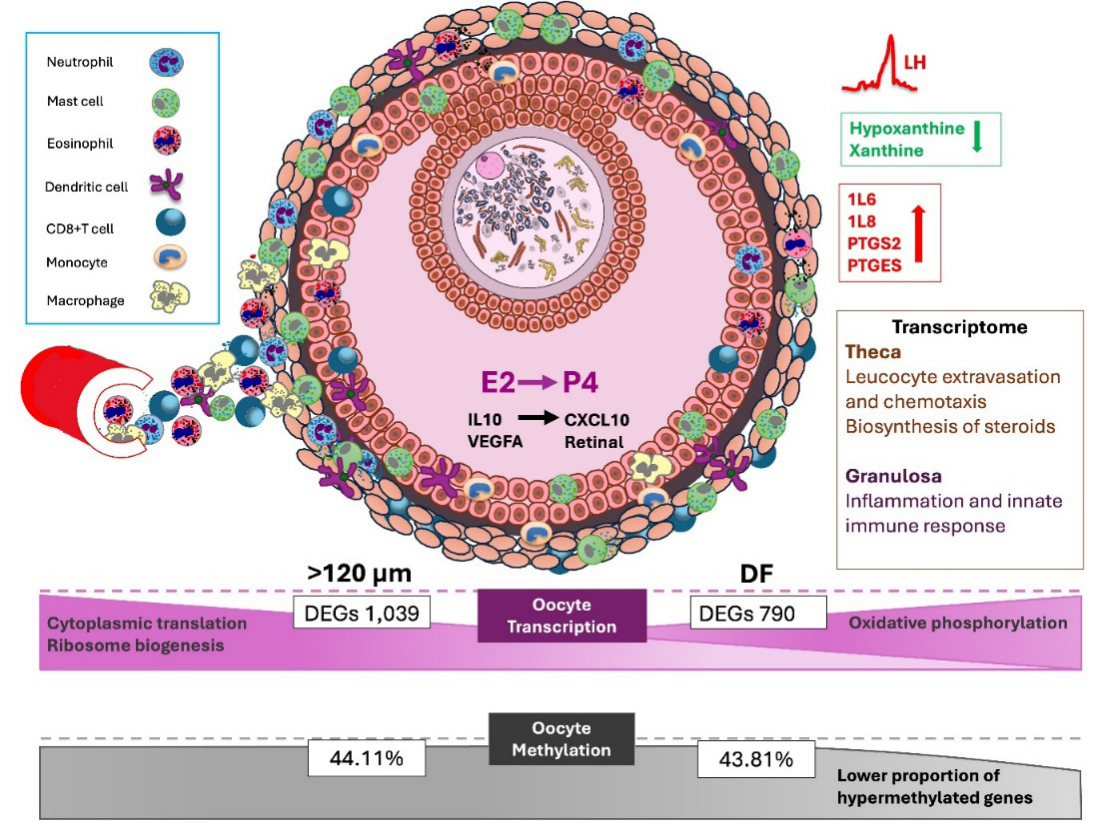
Summary diagram synthesizing the key changes in the microenvironment of the Dominant/Preovulatory follicle: Following the LH surge, there is a switch from high E2 to high P4 concentration in the follicular fluid. Increased blood flow into the follicle facilitates immune cell infiltration, a broad array of leukocytes are detected first within the theca layer and in the granulosa following the degradation of the basement membrane. Neutrophils, mast cells and dendritic cells are present in abundance and their secretions attract more immune cells to the follicle. The cytokines IL10 and VEGFA are highly concentrated in the follicular fluid 24 at the time of the LH surge, closer to the time of ovulation, their concentration decreases as CXCL10 increases. Similarly, the oocyte meiosis inhibitor metabolites Hypoxanthine and Xanthine are highly concentrated in the Follicular fluid at the time of the LH surge, but have decreased to nadir prior to ovulation, whereas prostaglandins PTGS2 and PTGES are undetectable at the time of the LH surge and are highly concentrated in the follicular fluid just prior to ovulation.

As summarized in Figure 6, these integrated hormonal, metabolic, immune, and molecular processes collectively govern follicle development, ovulation, and oocyte quality. However, they may be altered by health and metabolic challenges. Furthermore, discrepancies between our results and previous reports highlight the potential effects of common ARTs applied in cattle production, such as FSH stimulation and *in vitro* maturation, on the molecular integrity of oocytes, with implications for reproductive success in cattle.

The changing environment is reflected in the transcriptome of the theca and granulosa, genes within the biosynthesis of steroids pathway and genes associated with immune pathways related to leucocyte extravasation and chemotaxis are over-represented in theca cells, whereas immune pathways related to inflammation and innate immune response are over-represented in granulosa cells. As the oocyte within the dominant follicle has completed it’s growth phase, it is no longer transcriptionally active, therefore changes in gene expression are modest, but marked by an emphasis on mitochondrial function and oxidative phosphorylation. Similarly, changes in the global methylation profile are minor, however, the proportion of hypermethylated regions is reduced.

## Data Availability

Research data is available in a repository: GEO accession GSE297927.

## Supplementary Material

Supplementary material accompanies this paper:

Supplementary File 1: Material and methods.

Supplementary Table S1: scRNA seq sample quality control analysis performed in Rstudio by selecting samples with more than 100,000 reads and 2,500 expressed genes (genes with more than 1 count). Additional information is from MuitQC Report. The table on the right summarizes the information by experimental groups.

Supplementary Table S2: Differentially expressed genes between >120 um and DF oocytes. Significant genes were determined by FDR<0.05 and logFC>1.5. Enriched pathways from gene ontology analysis are presented in the panels on the right.

Supplementary Table S3: Sequencing output for DNA libraries from bovine final samples.

This material is available as part of the online article from: https://doi.org/10.1590/1984-3143-AR2025-0071
