# Supplementary material for: The dominant follicle: the final frontier in bovine oocyte development
Source: Anim Reprod. 2025 Aug 18;22(3):e20250071. doi: 10.1590/1984-3143-AR2025-0071 (PMC12490830; doi:10.1590/1984-3143-AR2025-0071)
Supplement: Supplementary File 1: [file 1984-3143-ar-22-3-e20250071-suppl01.pdf]

Material and methods

Animals

Oocyte samples were collected as part of two earlier trials performed at University College Dublin (Abdulrahman Alrabiah et al., 2021; Sánchez et al., 2024). Each animal trial was reviewed and approved by the Animal Research Ethics Committee of University College Dublin, Ireland, and licensed by the Health Products Regulatory Authority (HPRA), Ireland, in accordance with Statutory Instrument No. 543 of 2012 (under Directive 2010/63/EU on the Protection of Animals used for Scientific Purposes).

Oestrus synchronization protocol

At day zero (D0), an intramuscular injection of gonadotrophin-releasing hormone (GnRH) analogue (2 mL of Ovarelin – 50 ug/mL) was administrated to heifers, together with the insertion of P4-releasing intravaginal device (PRID). After 7 days (D7), corpus luteum regression was induced with the administration of prostaglandin F2a (PGF2a) analogue (5 mL of Enzaprost – 5 mg/mL), followed by PRID removal on day 8 (D8). Estrus detection was performed every 6h on day 8 (D8) and day 9 (D9). A second dose of GnRH was administered to a subgroup of animals 36 h after PRID removal (D9) to ensure a luteinising hormone (LH) surge. Animals were slaughtered on day 9 (24 h before ovulation) and on day 10 (19-23h after estrus detection and ~2h before ovulation – subgroup receiving the second GnRH). The uterine tract was recovered from each individual animal and transported to the laboratory on ice. The ovaries were isolated from the uterine tract, and the oocytes from the dominant follicles were collected by dissecting the follicle and searching for the oocyte under the stereomicroscope. Recovered oocytes were washed in PBS + 2% BSA, denuded from the cumulus cells by pipetting, and snap-frozen in liquid nitrogen in PCR microtubes (0.5 mL) before storing at -80 °C for sequencing analysis.

Single-cell RNA sequencing (scRNA-seq)

Oocyte samples were lysed by adding 8 μL of RTL-Plus buffer (Qiagen) to microtubes containing snap-frozen samples before transferring them to a 96-well plate for RNA and DNA isolation, as described by (Castillo-Fernandez et al., 2020). Briefly, oocyte lysates were incubated with Dynabeads (MyOne Streptavidin C1, Life

Technologies) annealed to Smart-seq2 oligo-dTs to capture polyadenylated mRNA. The remaining lysate containing the DNA was transferred to a new 96 well-plate for a subsequent bisulphite conversion analysis. The beads containing the mRNA were diluted in reverse transcription master mix using SuperScriptTM II Reverse Transcriptase (Invitrogen). cDNA was amplified (14 cycles) using KAPA HiFi HotStart ReadyMix (Roche), purified with Ampure XP beads, and eluted into 20 µL of water. Amplified cDNA was quantified using a High Sensitivity DNA Assay chip (Agilent Technologies), and libraries were prepared using the Nextera XT Kit (Illumina) with ~300 pg of cDNA. The individual transcriptome of 15 oocytes was sequenced on the NextSeq500 HighOutput 75 bp Single End with a sequence depth of ~2 million reads.

Single-cell bisulphite conversion (scBS-seq)

Oocyte DNA lysate transferred to the second 96-well plate was processed for bisulphite library preparation as previously described (Galvão & Kelsey, 2021). Briefly, bisulphite conversion was performed using EZ-96 DNA Methylation-Direct™ MagPrep (Zymo) according to the manufacturer's instructions. First-strand synthesis was performed by incubating converted DNA and mastermix for 3 min at 65 °C, followed by the addition of 1 µL of Klenow exo- (Enzymatics) and further incubation for 30 min at 37 °C. The process was repeated 4 times with the addition of 2.4 µL of the reaction mixture, followed by a final round of amplification at 37 °C for 90 min. Further, exonuclease treatment was performed for 1 hour at 37 °C, followed by purification with AMPure XP beads, resuspension in second-strand master mix, and incubation for 2 min at 98 °C. Klenow exo- (50U) was added to the mixture and incubated for 90 min at 37 °C. After purification, the libraries were amplified with 50 µL KAPA HiFi HotStart PCR Kit. Bisulphite conversion of 15 single oocytes was sequenced using NextSeq500 HighOutput 75 bp Paired End with a sequence depth of ~ 13 million unique reads.

Data processing and quality control

Raw sequence reads were trimmed using Trim Galore (version 0.6.7) (Krueger & Andrews, 2011) to remove adapter contamination and poor-quality reads with less than 20 Phred score, retaining reads with more than 20 bp sequence length for further

processing (Chhangawala et al., 2015; Williams et al., 2016). Data was mapped to the bovine reference genome (bosTau 9), and the genome index was generated using ARS-UCD1.2 (Rosen et al., 2020) assembly using HISAT2 (Kim et al., 2019) for RNA sequencing files, and Bismark (version 0.23.1) was used to align bisulphite sequence reads with Bowtie 2 (single-end non-directional) (Langmead & Salzberg, 2012) to the same bovine genome, and perform deduplication and methylation calls.

Data quantification and sample quality control were performed with SeqMonk software (version 11.0.10; Babraham Institute; https://www.bioinformatics.babraham.ac.uk/projects/seqmonk/) and RStudio software (2023.06.0+421). Using the SeqMonk RNAseq quality control plot, samples were identified and excluded due to a low number of counts falling inside genes. The remaining samples contained more than 100,000 reads and more than 2,500 expressed genes (genes with count number >1). A total of 21,218 transcripts were identified, and 15,975 remained after the exclusion of 5,243 non-expressed genes (genes with zero counts in all samples). A final sample set of 15 oocytes (14 >120 µm and 11 DF) and a dataset of 8,814 annotated genes were established for further bioinformatic analysis, performed with packages for the R software. Raw data from dominant follicle oocytes is deposited in the Gene Expression Omnibus repository and accessible through GEO accession number (GSE297927).

Similarly, quality control was performed in single-cell bisulphite sequencing dataset by excluding samples with less than 1 million CpGs covered and more than 10% non-CpG methylation (CHH and CHG), resulting in a final dataset from 21 single oocyte samples (12 >120 µm and 9 DF oocytes) (Supplementary Table S1 and S2).

Data statistical analysis

Sample distribution from scRNA-seq was visualized with PCA (Principal Component Analysis) and t-SNE (t-distributed Stochastic Neighbour Embedding), and sample variation was evaluated by a heat map of the sample-to-sample distances. Differential expression analysis was performed by comparing the two different groups using EdgeR package (Robinson et al., 2010), and adjusted p-values were calculated using Benjamini & Hochberg (Benjamini et al., 2001). The differently expressed genes (DEGs) were determined by those with a false discovery rate (FDR)<0.05 and log fold

change (logFC)>1.5. Gene Ontology (GO) enrichment analysis was performed using the enrichGO function from the clusterProfiler R package, focusing on the Biological Process (BP) ontology. The full set of DEGs was used as the background (universe). Terms with an adjusted p-value (p.adjust)<0.05 and q-value < 0.10 were considered significantly enriched (Supplementary Table S3).

Bisulphite quantification was initially performed over non-overlapping 50-CpG windows using the >120 μm group as a reference, including at least 1 cytosine within 5 consecutive cytosines per window. Only informative windows for both groups were considered for further analysis, with a total of 272,815 windows. Methylation pattern was determined as the percentage of CpG windows with 0-20, 20-40, 40-60, 60-80, and 80-100% methylation. Furthermore, hypermethylated (>75%) and hypomethylated (<25%) regions were also identified in each group. Secondly, methylation was quantified over genomic features, such as CpG islands, intergenic regions, gene promoters (1500 bp upstream and 500 bp downstream of transcription start sites), gene bodies, and transposable elements (long interspersed nuclear elements (LINE), short interspersed nuclear elements (SINE), and long-terminal repeats (LTR)) using previous annotations tracks (Ivanova et al., 2020).

REFERENCES

Benjamini, Y., Drai, D., Elmer, G., Kafkafi, N., & Golani, I. (2001). Controlling the false discovery rate in behavior genetics research. *Behav Brain Res*, *125*(1-2), 279-284. https://doi.org/10.1016/s0166-4328(01)00297-2

Castillo-Fernandez, J., Herrera-Puerta, E., Demond, H., Clark, S. J., Hanna, C. W., Hemberger, M., & Kelsey, G. (2020). Increased transcriptome variation and localised DNA methylation changes in oocytes from aged mice revealed by parallel single-cell analysis. *Aging Cell*, *19*(12), e13278. https://doi.org/10.1111/acel.13278

Chhangawala, S., Rudy, G., Mason, C. E., & Rosenfeld, J. A. (2015). The impact of read length on quantification of differentially expressed genes and splice junction detection. *Genome Biol*, *16*(1), 131. https://doi.org/10.1186/s13059-015-0697-y

Galvão, A., & Kelsey, G. (2021). Profiling DNA Methylation Genome-Wide in Single Cells. *Methods Mol Biol*, *2214*, 221-240. https://doi.org/10.1007/978-1-0716-0958-3_15

Kim, D., Paggi, J. M., Park, C., Bennett, C., & Salzberg, S. L. (2019). Graph-based genome alignment and genotyping with HISAT2 and HISAT-genotype. *Nat Biotechnol*, *37*(8), 907-915. https://doi.org/10.1038/s41587-019-0201-4

Krueger, F., & Andrews, S. R. (2011). Bismark: a flexible aligner and methylation caller for Bisulfite-Seq applications. *Bioinformatics*, *27*(11), 1571-1572. https://doi.org/10.1093/bioinformatics/btr167

Langmead, B., & Salzberg, S. L. (2012). Fast gapped-read alignment with Bowtie 2. *Nat Methods*, *9*(4), 357-359. https://doi.org/10.1038/nmeth.1923

Robinson, M. D., McCarthy, D. J., & Smyth, G. K. (2010). edgeR: a Bioconductor package for differential expression analysis of digital gene expression data. *Bioinformatics*, *26*(1), 139-140. https://doi.org/10.1093/bioinformatics/btp616

Rosen, B. D., Bickhart, D. M., Schnabel, R. D., Koren, S., Elsik, C. G., Tseng, E., Rowan, T. N., Low, W. Y., Zimin, A., Couldrey, C., Hall, R., Li, W., Rhie, A., Ghurye, J., McKay, S. D., Thibaud-Nissen, F., Hoffman, J., Murdoch, B. M., Snelling, W. M.,...Medrano, J. F. (2020). De novo assembly of the cattle reference genome with single-molecule sequencing. *Gigascience*, *9*(3). https://doi.org/10.1093/gigascience/giaa021

Williams, C. R., Baccarella, A., Parrish, J. Z., & Kim, C. C. (2016). Trimming of sequence reads alters RNA-Seq gene expression estimates. *BMC Bioinformatics*, *17*, 103. https://doi.org/10.1186/s12859-016-0956-2
